# Availability, cost, and budget impact of lifesaving postpartum hemorrhage interventions in public hospitals in Zambia: A cross-sectional survey

**DOI:** 10.1002/ijgo.70812

**Published:** 2026-02-02

**Authors:** Herbert Kapesa, Albert Manasyan, Nobutu Muttau, Rachel G. Sinkey, Ioannis Gallos, Tannia Tembo

**Affiliations:** 1 Reproductive, Maternal, Newborn, and Child Health, Centre for Infectious Disease Research in Zambia, Lusaka, Zambia; 2 Department of Pediatrics, University of Alabama at Birmingham, Birmingham, Alabama, USA; 3 ZAMBART, Social Science Department, Lusaka, Zambia; 4 Department of Obstetrics and Gynecology, University of Alabama at Birmingham, Birmingham, Alabama, USA; 5 Maternal and Perinatal Health Unit, World Health Organization, Geneva, Switzerland

**Keywords:** postpartum hemorrhage, tranexamic acid, uterine balloon tamponade, uterotonics

## Abstract

**Objective:**

Postpartum hemorrhage (PPH) is the leading cause of maternal mortality globally. Most PPH deaths are preventable through evidence-based interventions. This study assessed the availability, direct costs, and economic implications of World Health Organization-recommended PPH interventions in public hospitals in Zambia.

**Methods:**

A cross-sectional survey was conducted in 31 purposively selected public hospitals across seven provinces. Data (June 2019 to May 2020) on PPH prevalence, resource availability, and direct costs of PPH interventions were collected via a structured questionnaire administered to hospital administrators. An ingredients-based costing model compared an ideal pathway (severe PPH managed at a fully equipped primary-level health facility) to a referral pathway (patients unresponsive to early-stage interventions transferred to a tertiary hospital). Data were analyzed using Python.

**Results:**

Among 74 238 deliveries, 1957 (2.6%) were PPH cases, resulting in 94 (4.8%) fatalities. Most cases (86.9%) received medical management, primarily by nurse-midwives. Tranexamic acid (TXA) was available in only 58.1% of the hospitals (subsidized cost: US$1.91/dose). Managing a severe PPH case in the ideal pathway cost US$133.46–US$276.22, compared to US$153.34-US$332.53 via the referral pathway , representing an 18.6% increase. Scaled nationally (approximately 672 000 births), this inefficiency translates to an avoidable annual burden of US$133121.

**Conclusion:**

While oxytocin is universally available, access to critical treatments such as TXA and advanced interventions remains limited. The low cost of preventive interventions contrasts with the high cost of managing severe PPH, which is exacerbated by system fragmentation. Strengthening primary-level facilities with essential commodities, surgical capacity, and training is critical to containing costs and reducing maternal mortality due to PPH.

## 1 | INTRODUCTION

Globally, approximately 14 million women experience postpartum hemorrhage (PPH) each year, resulting in approximately 70 000 maternal deaths every year.^1^ In Zambia, PPH accounts for 34% of maternal deaths.^2^ Most PPH cases do not have identifiable risk factors, but those that predispose women to PPH can be categorized into antenatal, intrapartum, and postpartum. Over 80% of cases are due to uterine atony bleeding, followed by retained placenta tissue and uterine rupture.^3,4^

Most deaths are preventable through timely, evidence-based interventions, including uterotonics, uterine massage, tranexamic acid (TXA), uterine balloon tamponade (UBT), and surgical care. Prophylactic oxytocin is the mainstay for PPH prevention, while misoprostol and alternative injectable uterotonics are recommended when oxytocin is unavailable. TXA and UBT can be used to treat women who have not responded to early-stage treatment.^5^

The Zambian Ministry of Health (MoH) has adopted the World Health Organization (WHO)-recommended PPH prevention and treatment protocols for all health facilities offering labor and delivery services.^6^ Given the critical importance of timely interventions, assessing health facility readiness and cost is critical for effective implementation. This study evaluated the availability, direct costs, and economic implications of WHO-recommended PPH interventions in public hospitals in Zambia.

## 2 | MATERIALS AND METHODS

### 2.1 | Study design and setting

A facility-level, descriptive cross-sectional survey was conducted in government and mission hospitals in Zambia between November 1 and December 15, 2020, to assess PPH service availability from June 2019 to May 2020. Hospitals were selected based on annual delivery volume (500–5000 births). Government hospitals are fully publicly funded, while mission hospitals receive co-funding from the government and churches.

### 2.2 | Data sources and collection methods

A validated questionnaire, adapted from a multi-country PPH trial assessment tool, was administered by a trained nurse-midwife to hospital administrators via in-person interviews for health facilities in Lusaka Province and via telephone for those in other provinces, in compliance with COVID-19 protocols.^7^ The questionnaire collected data on the availability of staff, medications, supplies, PPH protocols, blood transfusion services, and surgical and intensive care capacity. Data on PPH cases and outcomes and commodities were extracted from labor and delivery and referral registers, pharmacy inventories, and official procurement records. PPH cases, outcomes, and costs were extracted from registers, pharmacy inventories, and official procurement records.

### 2.3 | Postpartum hemorrhage guidelines and clinical workforce in Zambia

Zambia’s PPH guidelines align with WHO recommendations and promote a bundle of interventions, including uterotonics, uterine massage, UBT, and timely escalation to advanced care if bleeding persists. Care is provided at no cost in public health facilities. The clinical hierarchy comprises medical doctors, clinical officers (3-year diploma holders), and midwives. Doctors perform major procedures mainly at tertiary-level facilities, while clinical officers and midwives are frontline providers at primary-level facilities. Intern doctors work under supervision. The protocol, spanning preventive measures and management of severe PPH, costs of commodities, and personnel time, is outlined in Table 1.

### 2.4 | Costing consider framework and assumptions

An ingredient-based micro-costing model was developed to estimate the direct cost of guideline-compliant PPH management, structured around the staged clinical pathways outlined in the Zambian national PPH guidelines. The model simulated two distinct clinical scenarios (refer to Table 2 for key assumptions): (i) an ideal pathway, a fully equipped primary-level facility; and (ii) referral patients stabilized at a primary-level facility and transferred to a tertiary hospital for advanced care. Costs included medications, consumables, laboratory tests, personnel, and ambulance transfer. Capital, overhead, and patient out-of-pocket costs were excluded. This base-case model calculated the cost for each PPH stage independently and did not consider probabilistic progression rates between stages.

Unit costs were sourced from the Zambia Medicines Supplies Agency (ZAMMSA) price list and converted using the 2020 exchange rate (US$1 = ZMW 16.10). Personnel costs were based on government salary scales with task time estimates provided by clinical experts. Table 1 details the unit and cumulative costs for each intervention stage. Costs per case were extrapolated using the observed 2.6% PPH incidence to a hypothetical cohort and a national estimate of 672 000 annual births.^8^

### 2.5 | Data analysis

Data were analyzed in Python. Descriptive statistics summarized hospital characteristics, resource availability, and outcomes. Comparative costs for the two pathways were calculated. A one-way sensitivity analysis varied key parameters (±20%) to identify key cost drivers and assess the robustness of the cost estimate for severe PPH management.

### 2.6 | Ethical considerations

Ethical clearance was obtained from the University of Zambia Biomedical Research Ethics Committee (UNZABREC—#001-07-20) and the National Health Research Authority (NHRA). Informed consent was waived as no patient data were collected.

## 3 | RESULTS

### 3.1 | Deliveries and postpartum hemorrhage outcomes

Of the 74 238 deliveries, 10 704 (14.4%) were cesarean sections. There were 1957 (2.6%) PPH cases, 94 (4.8%) of which resulted in fatalities. Rural faith-based facilities contributed nearly half (48.9%) of all referrals to urban facilities. Most cases (1086; 86.9%) received medical management, while 164 (13.1%) underwent laparotomy (Table 3). Interventions were not mutually exclusive, as some patients were managed with multiple interventions.

### 3.2 | Health facility characteristics

The survey included data from 31 public hospitals across seven provinces in Zambia. Guidelines for PPH care were available in 28 hospitals (90.3%). Oxytocin was universally available, while TXA was available in only 18 (58.1%) of facilities. Intensive care units were present in only two facilities (6.5%). Surgical capacity for laparotomies was present at 26 hospitals (83.9%), and blood transfusion services were available in all but one (96.8%) facility (Table 4).

### 3.3 | Healthcare workforce

**FIGURE 1:**
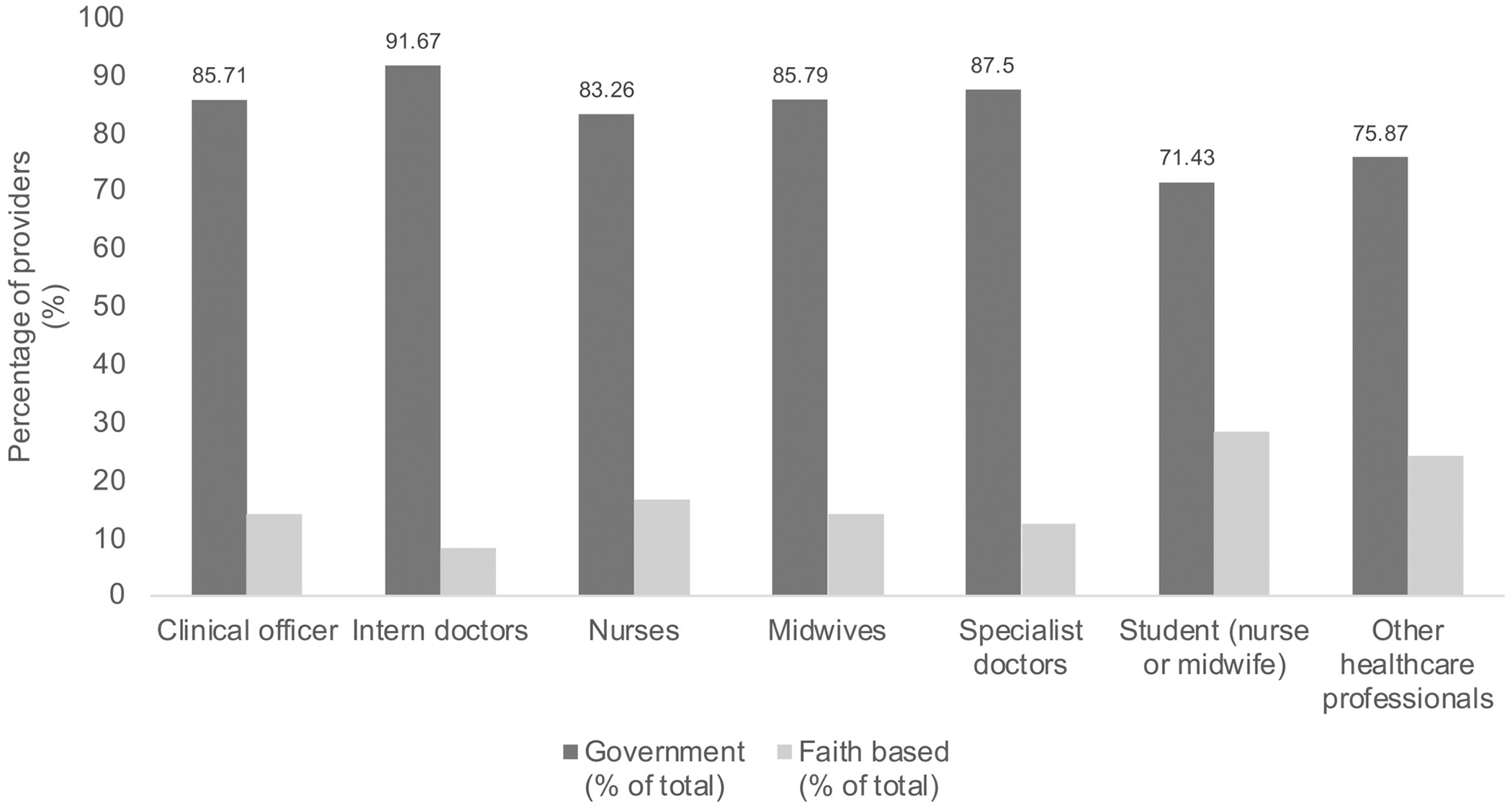
Healthcare worker distribution by health facility type.

Of the 878 healthcare providers at the surveyed health facilities, midwives (41.7%) and nurses (29.3%) constituted the majority. Specialist doctors (0.9%) and medical interns (10.2%) were scarce. In contrast, clinical officers were evenly distributed between rural and urban health facilities. Staff distribution favored urban hospitals, with midwives and nurses forming the bulk of the workforce (Figure 1).

### 3.4 | Cost implications of clinical pathways and facility capability

**FIGURE 2:**
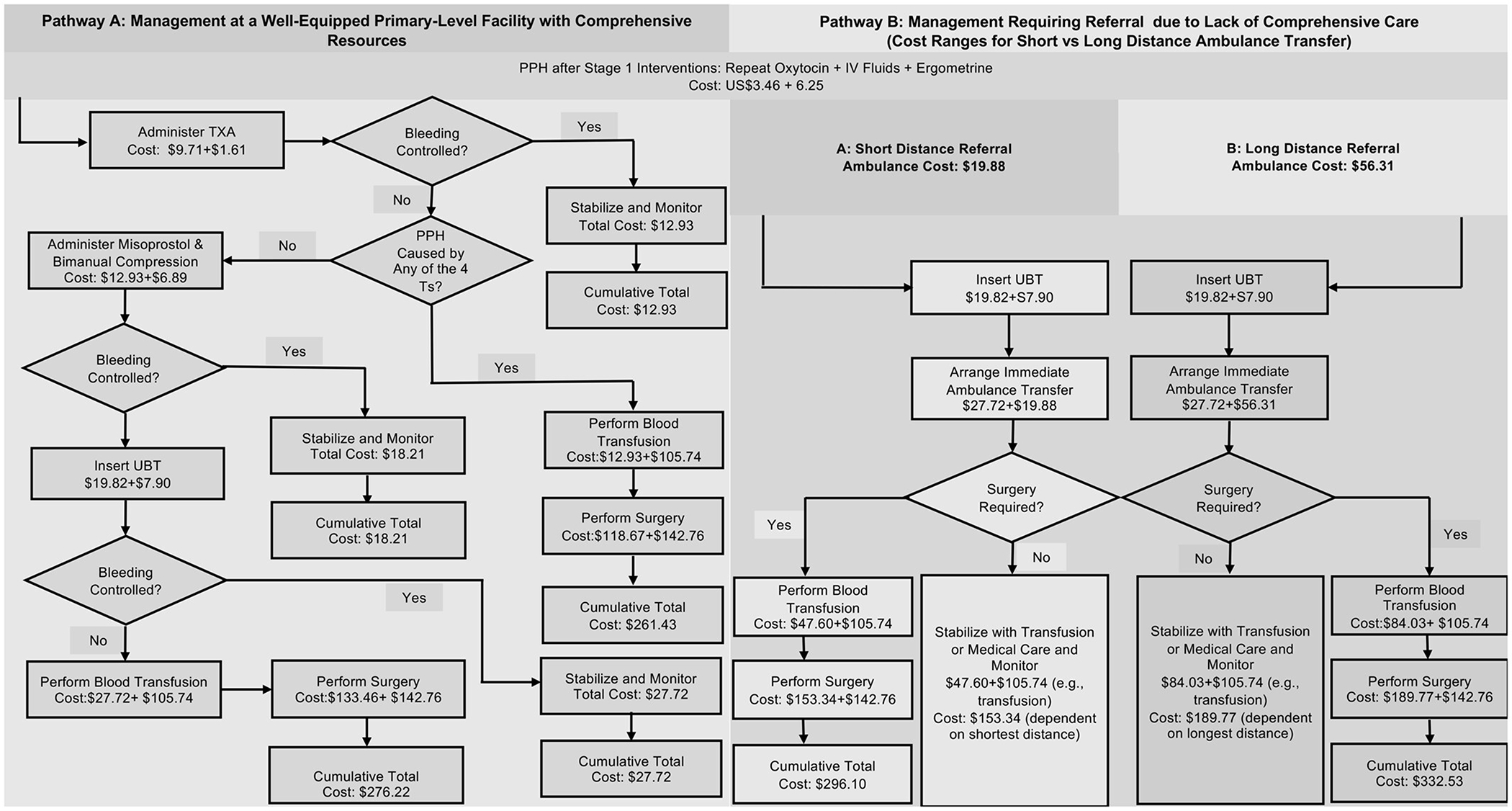
Cost comparison of clinical pathways for postpartum hemorrhage (PPH) management: Ideal versus referral scenarios. IV,intravenous; TXA, tranexamic acid.

Figure 2 illustrates the economic impact of facility capability and geographic access through two clinical pathways for managing severe PPH following initial uterotonic failure. The ideal pathway (comprehensive on-site management at a well-equipped primary-level facility) shows the sequential cost escalation from a baseline of US$9.71, up to US$133.46 with blood transfusion and US$276.22 with surgery. Refractory hemorrhage attributed to the “4 Ts” (tissue, thrombin, tone, and trauma) requires advanced care immediately after the administration of TXA and costs US$118.67 when a unit of blood is transfused and US$261.43 when surgery is performed. The referral pathway (fragmented care requiring referral) includes transfer costs and results in total costs ranging from US$153.34 (short-distance transfer + transfusion) to US$332.53 (long-distance transfer + surgery + transfusion).

### 3.5 | Budget impact analysis

Scaling the analysis to Zambia’s estimated 672 000 annual births reveals a substantial national financial impact (Table 5). Based on the observed PPH incidence of 2.6%, an estimated 3494 severe cases are projected to occur annually. Managing all severe cases via the ideal pathway would cost US$715711 nationally, compared to US$848832 under the current-referral-dependent system, an avoidable burden of US$133121 per year, representing the same 18.6% cost increase observed per case.

### 3.6 | Sensitivity analysis and key cost drivers

**FIGURE 3:**
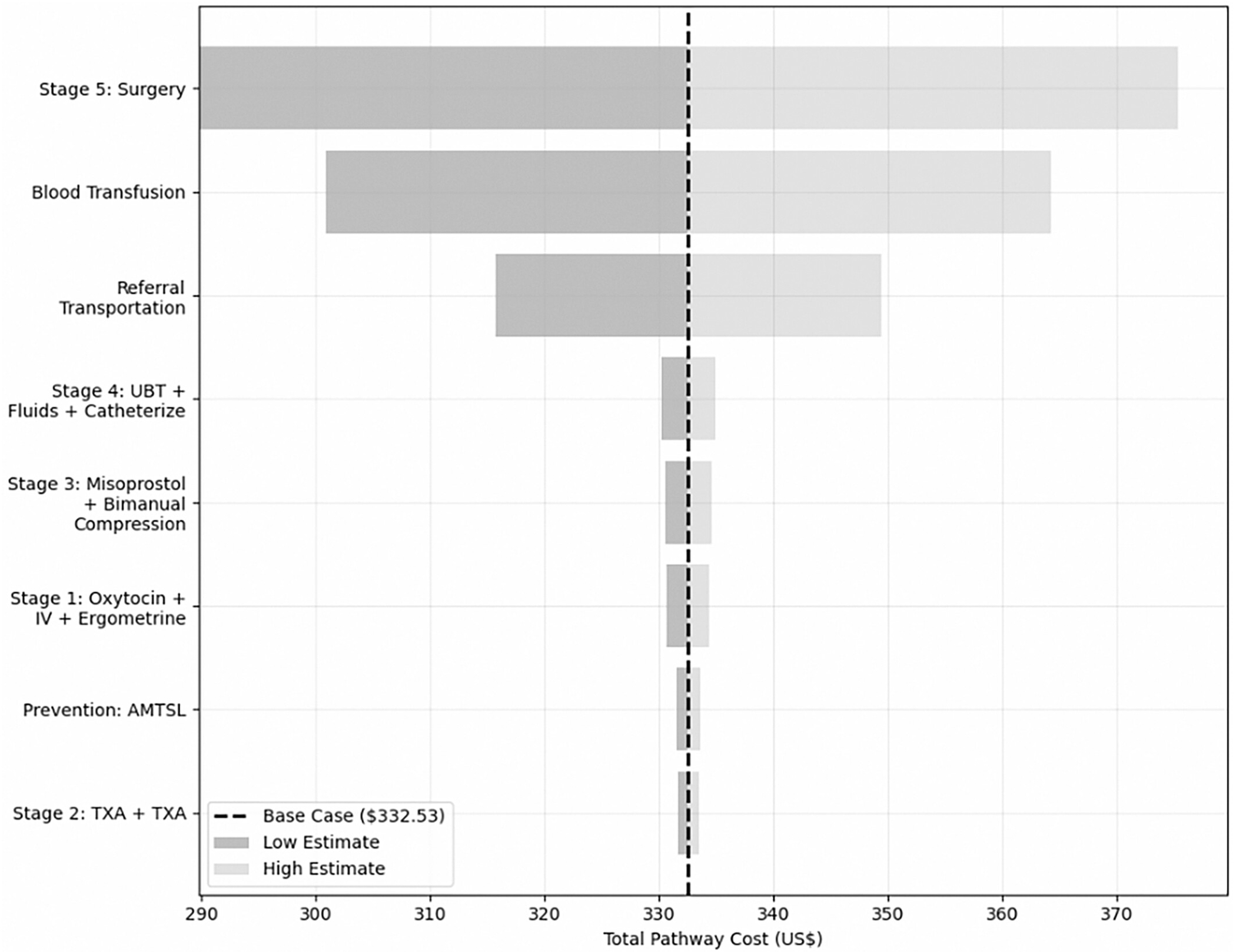
Drivers of cost variability in postpartum hemorrhage (PPH) treatment pathways. IV, intravenous; TXA, tranexamic acid.

Sensitivity analysis (Figure 3) identified the key drivers of cost variability in the management of severe PPH under the referral pathway. Parameters were varied by ±20% and measured its impact on total pathway cost, with a base case estimate of cost of US$332.53. Surgery and blood transfusion were the primary cost drivers, each exerting the greatest influence on the total pathway cost. Referral transportation was the third most influential variable. In contrast, costs associated with preventive measures (e.g., active management of the third stage of labor) and early-stage interventions (stage 1: oxytocin, IV, and ergometrine; stage 2: TXA + TXA) had minimal impact on overall cost outcomes.

## 4 | DISCUSSION

This study reveals a high PPH incidence and significant referral rates from primary-level facilities, indicating an overdependency on tertiary-level hospitals for managing obstetric emergencies. These findings underscore a critical gap in the continuum of care at the most basic level of care, likely driven by stockouts of essential commodities, insufficiently trained staff, and poorly defined protocols. There is an urgent need to strengthen basic PPH management capabilities through reliable supply chains, targeted training, and clear context-specific protocols.

Facility readiness and access to commodities varied widely. Contrary to a 2017 multi-country assessment,^9^ our findings confirm improved availability of subsidized uterotonics like oxytocin. However, access to critical advanced interventions remained constrained. A notable shortage was observed for TXA, which was available in only 58.1% of facilities despite its demonstrated cost-effectiveness in reducing mortality when administered early.^10,11^ The UBT, recommended for stabilizing patients before transfer if initial and subsequent treatments fail, was utilized in less than 1% of PPH cases in this study. This low utilization suggests significant gaps in device availability, reinforcement of training, or provider confidence.^12–14^

The skewed distribution of healthcare workers, with a concentration of doctors in urban areas and a reliance on midwives and nurses in rural facilities, exacerbates disparities in emergency obstetric care. This aligns with patterns in Kenya, Nigeria, and South Africa.^15,16^ While most facilities had PPH guidelines, their consistent application might be hindered by accessibility issues. Digital distribution of protocols via official platforms could improve adherence.^17,18^

This costing analysis established a benchmark for PPH management. Preventive active management of the third stage of labour cost US$1.96 (US$3.46 per personnel), aligning with other low-resource settings.^19^ Similarly, subsidized TXA (US$1.91 per dose in India; US$1.61 in Zambia) reflected the substantial influence of government-negotiated procurement in keeping essential commodities financially accessible.^20^ However, these subsidized costs likely understate the true economic expenses, as they include donor contributions.^21^ In contrast, the economic burden of managing severe PPH escalates dramatically and is significantly influenced by the level of facility readiness and the clinical pathway followed. Additionally, costs for advanced care are primarily driven by blood transfusion and surgery, a finding consistent with economic analyses from Egypt.^22^

The cost comparison between the ideal pathway (comprehensive management at a well-equipped primary-level facility) and the referral pathway (transfer to a tertiary hospital) reveals a steep rise in cumulative costs under the referral scenario, driven by ambulance transfer, repeated interventions, and delayed access to advanced care. This quantifies the financial inefficiency inherent in fragmented systems^23,24^ and underscores that investing in primary-level capacity is both a clinical and economic priority. For policymakers, the ideal pathway cost represents the investment needed for a functional PPH response system.

Scaling our facility-level costing nationally reveals the substantial financial consequences of fragmented PPH care in Zambia. Based on an estimated 672 000 annual births and a PPH incidence of 2.6%, we project 17 472 PPH cases yearly and 3494 (20%) severe cases. Managing all severe cases via the ideal pathway would cost US$715711 annually, compared to US$848832 under the current referral-dependent system, an avoidable US$133121 per year (18.6% increase) due to system fragmentation. Investing in essential commodities, surgical capacity, and training at primary-level facilities could prevent this economic waste while improving maternal outcomes and equity in emergency obstetric care.

The findings represent a snapshot from 2020 during the COVID-19 pandemic, and service availability might have since evolved. Pandemic-related constraints, including travel restrictions, staff redeployment, and facility access issues, likely affected data completeness and may introduce bias, particularly in rural areas. Further, the cost estimates may be influenced by pandemic-era price volatility, which could affect the accuracy of budgetary projections. Communication and documentation during referral were consistently poor, directly impeding the tracking of referral outcomes and resource utilization. This limited our ability to fully capture the clinical and cost trajectories of referral pathways, a gap that likely stems from systemic communication failures between facilities. Such failures undermine continuity of care, delay clinical decision-making, and obscure the true outcomes, complications, and complicate accurate economic modeling of the referral pathway.

Additionally, this costing analysis excluded indirect and capital expenses, thus presenting a lower-bound estimate of the financial burden. The absence of individual-level data restrcited our analysis of referral reasons, patient-level cost drivers, and some clinical outcomes. Finally, provider competency was not assessed. Despite these gaps, this study provides novel, Zambia-specific evidence on the availability and direct costs of PPH services in public health facilities, offering critical data for assessing progress toward implementing global guidelines such as those from the WHO. It identifies key service gaps and urban–rural disparities, equipping policymakers with actionable insights for targeted resource allocation in seven of Zambia’s 10 provinces. Further, the cost data furnish an essential evidence base for future economic evaluations of PPH care.

## 5 | CONCLUSION

While PPH remains a leading cause of maternal mortality, systemic gaps pose a critical challenge to the delivery of appropriate and timely care. This study highlights significant gaps in the availability, accessibility, and cost of key WHO-recommended PPH interventions across public health hospitals in Zambia. The high PPH incidence and substantial referral rates from primary-level facilities underscore the challenges in managing obstetric emergencies. The disproportionate reliance on referrals, coupled with limited advanced interventions, suggests a need for improved capacity at lower-level facilities to manage PPH effectively. While basic interventions are relatively affordable, advanced interventions impose a significant financial burden on the healthcare system. Addressing the identified gaps is essential for reducing maternal mortality and achieving equitable, cost-effective PPH management in Zambia. Policymakers must prioritize strengthening the health system, developing the workforce, and ensuring sustainable financing to ensure equitable access to PPH services and sustainable gains in maternal health. Further, health facility assessments should be conducted periodically to help identify gaps and allocate resources effectively.

## Figures and Tables

**TABLE 1:** Unit and additive costs of postpartum hemorrhage (PPH) interventions from prevention to advanced care.

Stage	Intervention option	Description	Commodity unit cost (US$)	Personnel cost per hour (US$)	Total cost per intervention (US$)	Cumulative cost (US$)	Source	Notes
Prevention	Active management of third stage of labor	CCT + Oxytocin (10 IU) + Uterine massage	1.96	1.5	3.46	3.46	Güngördük, Olgac, and Kocaer, 2015	Routine care for all births to prevent PPH

Stage 1A (early management)	Repeat oxytocin + IV fluids	10 units IM+ 1000 mL IV fluids (normal saline or Ringer’s lactate)	1.53 0.9	1.65	4.08	7.54	ZAMMSA, 2023^8^	US$3.46 + 4.08—Represents a case where bleeding is controlled with uterotonics and fluids.
Stage 1B (early management)	Repeat oxytocin + IV fluids + ergometrine or synometrine	10 units IM + 1000 mL IV fluids + 0.5 mg IM/IV	1.97	0.20	2.17	9.71		US$7.54 + 2.71—Cost of stage 1A + ergometrine

Stage 2A (uncomplicated management)	TXA—1st dose	1 gm (10 mL) administered via slow IV injection	1.11	0.50	1.61	11.32	ZAMMSA, 2023^8^	US$9.71 + 1.61. Bleeding continues, requiring first-line TXA
Stage 2B (Uncomplicated Management)	TXA—2nd dose	2nd 1 g dose administered if bleeding persists	1.11	0.50	1.61	12.93		US$11.32 + 1.61. Bleeding persists. Second dose of TXA administered

Stage 3 (severe PPH at primary-level facility)	Misoprostol + Bimanual compression	1000 μg (5 × 200 μg tablets) sublingual/rectal + Bimanual compression of the uterus (done concurrently)	5.99	0.90	6.89	19.82	Escobar, et al., 2022^5^	US$12.93 + 6.89. For severe PPH management at primary-level facility. Misoprostol can be given as an alternative or in addition to TXA

Stage 4 (emergency transfer)	UBT + catheterize + fluids	Inflated foley catheter or condom with 500 mL + Normal saline	6.90	1.00	7.90	27.72	ZAMMSA, 2023	US$19.82 + 7.90. Case requires procedural intervention and transfer to a close by higher-level facility
	Ambulance transfer	Ambulance, fuel, allowances for driver and accompanying nurse-midwife	19.88–56.31	-	19.88	47.60		US$27.72 + 19.88. Case requires procedural intervention and transfer to a close by tertiary-level facility
	Ambulance transfer	Ambulance, fuel, allowances for driver and accompanying nurse-midwife	19.88–56.31	-	56.31	84.03		US$27.72 + 56.31. Case requires procedural intervention and transfer to a long distant tertiary-level facility

Stage 5A (advanced management at tertiary hospital)	Surgery	Laparotomy/hysterectomy, compression sutures, or arterial ligation	120.6	22.16	142.76	190.36	ZAMMSA, 2023^8^	US$47.60 + 142.76. Represents the full pathway for a patient requiring definitive surgical management. Used the lower bound estimate for ambulance transfer
			120.6	22.16	142.76	226.79		US$84.03 + 142.76. Represents the full pathway for a patient requiring definitive surgical management. Used the upper bound estimate for ambulance transfer
Stage 5B (advanced management at tertiary hospital)	Blood transfusion (1 unit)		100	5.74	105.74	296.10	Global Press Journal, 2023	US$190.36 + 105.74. Represents the full pathway for a patient requiring definitive surgical management and 1 unit of blood. Used the lower bound estimate for ambulance transfer
			100	5.74	105.74	332.53		US$47.60 + 142.76. Represents the full pathway for a patient requiring definitive surgical management and 1 unit of blood. Using the lower bound estimate for ambulance transfer

*Note*: All cost reflect direct costs only, covering commodities and personnel time based on task duration and salary scales. Capital and overhead costs are not included. Additive costs: Each scenario includes the costs of all preceeding interventions. Values represent the price per prescribed unit. Ambulance cost: Upper bound estimates were applied for ambulance transfers. Personnel costs: Calculations were based on government salary scales and the time-per-task. Annual leave, rural hardship allowances, overtime, and similar benefits are excluded. Supply assumptions: For stages offering multiple supplies (e.g. Normal Saline or Ringer’s Lactate), the model assumes the use of one unit. Surgery and transfusion costs: Stage 1 includes repeat oxytocin administration and IV fluids and/or ergometrine. Stage 2 includes the cost of a single dose of TXA; a repeat dose is itemized separately. Stage 5 shows two possible outcomes: surgery or blood transfusion. Surgical costs incoporate the combined salaries of multiple staff for a single patient procedure. Abbreviations: IV, intravenous; TXA, tranexamic acid.

**TABLE 2:** Key assumptions for clinical pathways for managing severe PPH cases.

Category	Assumption for severe PPH managed at a well-equipped primary-level facility	Assumption for severe PPH escalated to tertiary-level hospital	Rationale
Costing perspective^a^	Health system perspective (excluding indirect and patient costs)	Health system perspective (excluding indirect and patient costs)	Provides comprehensive view of financial burden and acknowledges practical data constraints while focusing on what the health system can directly finance and control
Facility level	Primary-level health center with adequate emergency obstetric resources^b^	Tertiary-level hospital with advanced surgical and critical care capacity^c^	Reflects Zambia’s tiered health system structure. Cost comparison at all levels provides a more accurate picture of the financial burden of a PPH case on the entire health system
Clinical pathway	Hemorrhage managed with first-line and second-line interventions. Proceed to advanced care if patient is non-responsive	Hemorrhage does not respond to initial interventions; case escalates to tertiary-level for advanced care^a^	Based on Zambia’s national PPH guidelines, which outline step-by-step management at each stage of care
Interventions	Repeat oxytocin (post AMTSL), IV fluids, uterine massage, monitoring; may include UBT if available. Perform surgery and perform blood transfusion if needed	Manage with UBT kit on transfer, drugs, surgical supplies for laparotomy, and/or blood transfusion if required	Models step-by-step care with escalating interventions aligned to severity while representing the resources utilized. Informs resource planning and strategic decision-making
UBT	Nurse-midwife (approximately 90 min of care) and/or clinical officer (approximately 20 min for decision-making on next steps of management)	Surgical team: obstetrician, anesthetist, theater nurses (approximately 3 h for surgery and postoperative care)	Aligns intervention with specific cadre, approximates the time required for patient management. Hourly rate based on Zambian public sector salaries for 2023/2024
Consumables and supplies	IV line, fluids, gloves, personal protective clothing, needles and syringes, etc.	All primary-level consumables plus surgical kit, sutures, anesthesia supplies, blood products	Costs accumulate across stages; severe cases that require escalation have an additional cost for ambulance transfer
Laboratory tests	None or full transfusion workup and transfusion monitoring tests	None or full transfusion workup and transfusion monitoring tests	Laboratory utilization is primarily low in uncomplicated PPH. However, laboratory services are essential for severe PPH cases, particularly if blood transfusion is required
Transport	Not required (assumes management at the same facility)	Emergency ambulance referral from primary- to tertiary-level hospital	Assumes escalation occurs only when severe PPH requires surgical or advanced interventions unavailable at primary level
Outcome	Hemorrhage controlled without need for referral; stabilization achieved on site	Case progresses to severe PPH requiring surgery; UBT used during transfer, and postoperative monitoring at tertiary-level hospital^d^	Enables clear costing by modeling two distinct, mutually exclusive care pathways

Abbreviations: AMTSL, active management of the third stage of labour; IV, intravenous; UBT, uterine balloon tamponade.

a Costs at each stage do not include indirect or out of pocket costs.

b Primary-level health facility—health center or district-level hospital with basic emergency obstetric and newborn care (BEmONC).

c Tertiary-level health facility—hospital with comprehensive emergency obstetric and newborn care (CEmONC) capacity.

d Severe PPH was defined as blood loss ≥1000 mL or bleeding associated with signs of hemodynamic instability requiring urgent intervention.

**TABLE 3:** Deliveries, PPH case management, and outcomes by facility location.

	Urban		Rural		
Facility ownership	MoH, *n* (% of total)	Faith based, *n* (% of total)	MoH, *n* (% of total)	Faith based, *n* (% of total)	Total
Deliveries					
Number of deliveries	54 695 (73.7)	706 (0.9)	13 130 (17.7)	5707 (7.7)	74 238
Number of cesarean-sections	7710 (72.0)	51 (0.5)	1942 (18.1)	1001 (9.4)	10 704
PPH cases					
Referrals^a^	257 (13.1)	9 (0.5)	735 (37.6)	956 (48.9)	1957
PPH management					
Treatment^b^	534 (53.6)	18 (1.8)	352 (35.3)	92 (9.2)	996
UBT	56 (62.2)	2 (2.2)	29 (32.2)	3 (3.3)	90
Laparotomy^c^	119 (72.6)	0 (0.0)	37 (22.6)	8 (4.9)	164
PPH death					
PPH deaths	53 (56.4)	7 (7.5)	25 (26.6)	9 (9.6)	94

Abbreviations: MoH, Ministry of Health; PPH, postpartum hemorrhage.

a Referrals show the number of cases received from lower-level facilities.

b Treatment provided using oxytocin, tranexamic acid, and fluids.

c Laparotomy includes hysterectomy, compression sutures, or arterial ligation.

**TABLE 4:** Characteristics of health facilities.

	Urban		Rural		
Facility ownership	MoH	Faith based	MoH	Faith based	Total, *n* (% of total)
Total facilities, *n* (% of total)	18 (58)	1 (3.2)	7 (22.6)	5 (16.2)	31

Resources					
PPH care guidelines	15	1	7	5	28 (90.3)
Oxytocics	18	1	7	5	31 (100.0)
Tranexamic acid	13	0	2	3	18 (58.1)
Blood transfusion	17	1	7	5	30 (96.8)
Intensive care unit	2	0	0	0	2 (6.5)

Services					
Routine use of oxytocics in the third stage of labor	18	1	7	5	31 (100.0)
Assisted vaginal delivery	18	1	7	5	31 (100.0)
Neonatal resuscitation	18	1	7	5	31 (100.0)
Maternal cardiopulmonary resuscitation	16	0	6	5	27 (87.1)
Hysterectomy	15	0	7	4	26 (83.9)

*Note*: MoH percentages are calculated out of the total number of health facilities surveyed (*N*n= 31). Resource and service availability reflect on-site capacity reported at the time of assessment and may vary in routine practice.

Abbreviation: PPH, postpartum hemorrhage, MoH, ministry of health.

**TABLE 5:** Budget impact analysis of severe PPH management pathways for Zambia.

Parameter	Value	Calculation/assumption
Total births	672 000	Annual deliveries based on national demographic data^a^
Total PPH cases	17 472	Based on the observed study incidence of 2.6%^b^
Severe PPH cases	3494	Assumed to be 20% of PPH cases require advanced care^c^
Management pathway	Average cost per severe case (US$)	Total pathway cost (US$)
Ideal pathway (managed at primary-level health facility)^d^	204.84	715710.96
Referral pathway (with transportation and allowances)^e^	242.94	848832.36
Cost difference financial burden due to referral	—	133121.40
Percentage Increase	—	18.6%

Abbreviation: PPH, postpartum hemorrhage.

a Birth estimate based on Zambia’s total fertility rate and population.

b Based on the observed PPH incidence of 2.6% from the study setting.

c Estimate for the proportion of PPH cases progressing to severe PPH requiring advanced intervention. Assumption: 20% of PPH cases progress to severe PPH requiring advanced intervention.

d Ideal pathway assumes all severe PPH cases are managed at a well-equipped primary-level facility with comprehensive resources. The average cost per case ($204.84) was derived from the midpoint of the modeled cost range (US$133.46–US$276.22) in the clinical pathway analysis.

e Referral pathway assumes severe PPH cases require transfer to a tertiary-level facility due to lack of essential interventions at the primary-level facility. The average cost per case ($204.84) was derived from the midpoint of the modeled cost range (US$153.34–US$332.53), inclusive of ambulance transfer costs.

## Data Availability

The data that support the findings of this study are available from the corresponding author upon reasonable request.
